# Supplementary material for: In Silico Protein Folding Prediction of COVID-19 Mutations and Variants
Source: Biomolecules. 2022 Nov 10;12(11):1665. doi: 10.3390/biom12111665 (PMC9688002; doi:10.3390/biom12111665)
Supplement: Supplementary file 1 [file biomolecules-12-01665-s001.zip › biomolecules-1969693-supplementary.pdf]

Supplementary Figures:

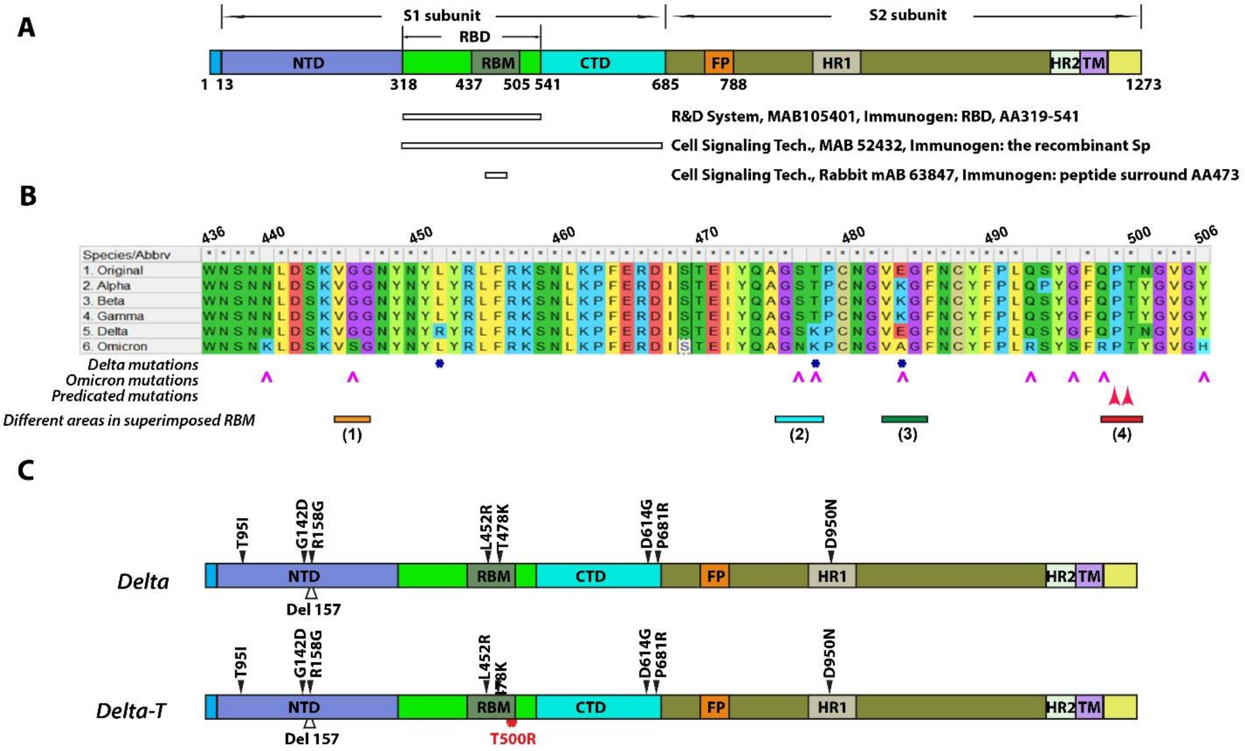

Supplementary Figure S1: Scheme of COVID-19 Spike protein. (A) Simple diagram of Spike protein. The three antibodies' recognized regions are shown. RBD: Receptor Binding Domain; RBM: ACE-2 Receptor Binding Motif; FP: furin process domain; (B) Amino acid alignment of RBM in all different known variants. (c) Spike mutation of Delta Variant was tagged with mREF in N terminal. T500R mutation was mutated in the RBM mutation

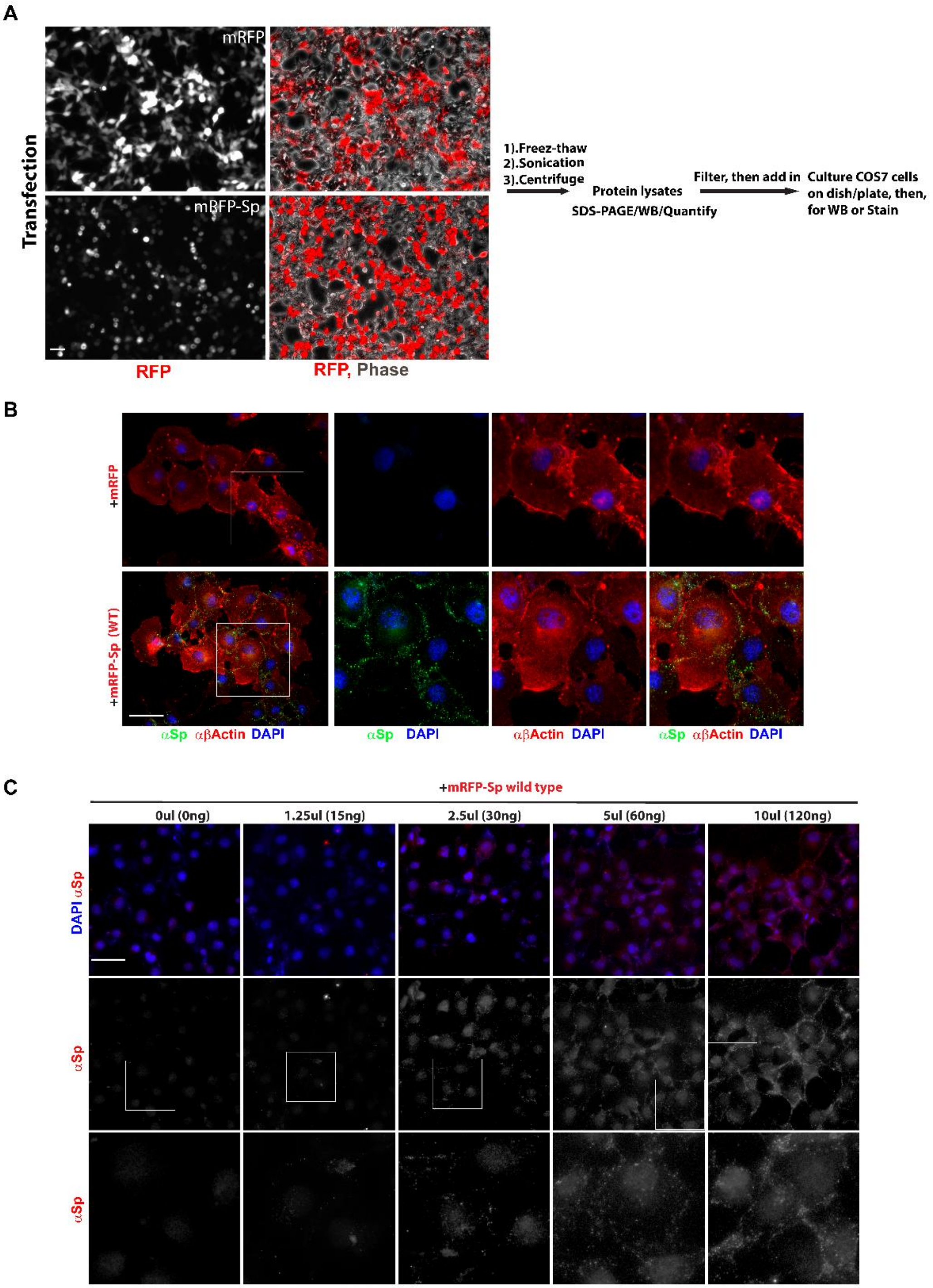

Supplementary Figure S2: mREF- Spike protein binds to cell membrane (A) Strategy of cell lysate preparation and ligand cell bound assay. 293T cells were transfected with plasmids for two days, then process cell lysis via freeze thaw method. After quantification with western blot the exact number of protein lysates were filtered and added to the pre-cultured COS 7cells. (B) mRFP-Sp protein was detected majority around cell surface either via RFP (data not shown) or Spike protein antibodies' staining. (C) Binding affinity by dosage. Different concentrations of spike protein lysates were added to measure the binding curve. Around 100 ng spike protein is reaching the station platform. Scale bar: 20 µm.

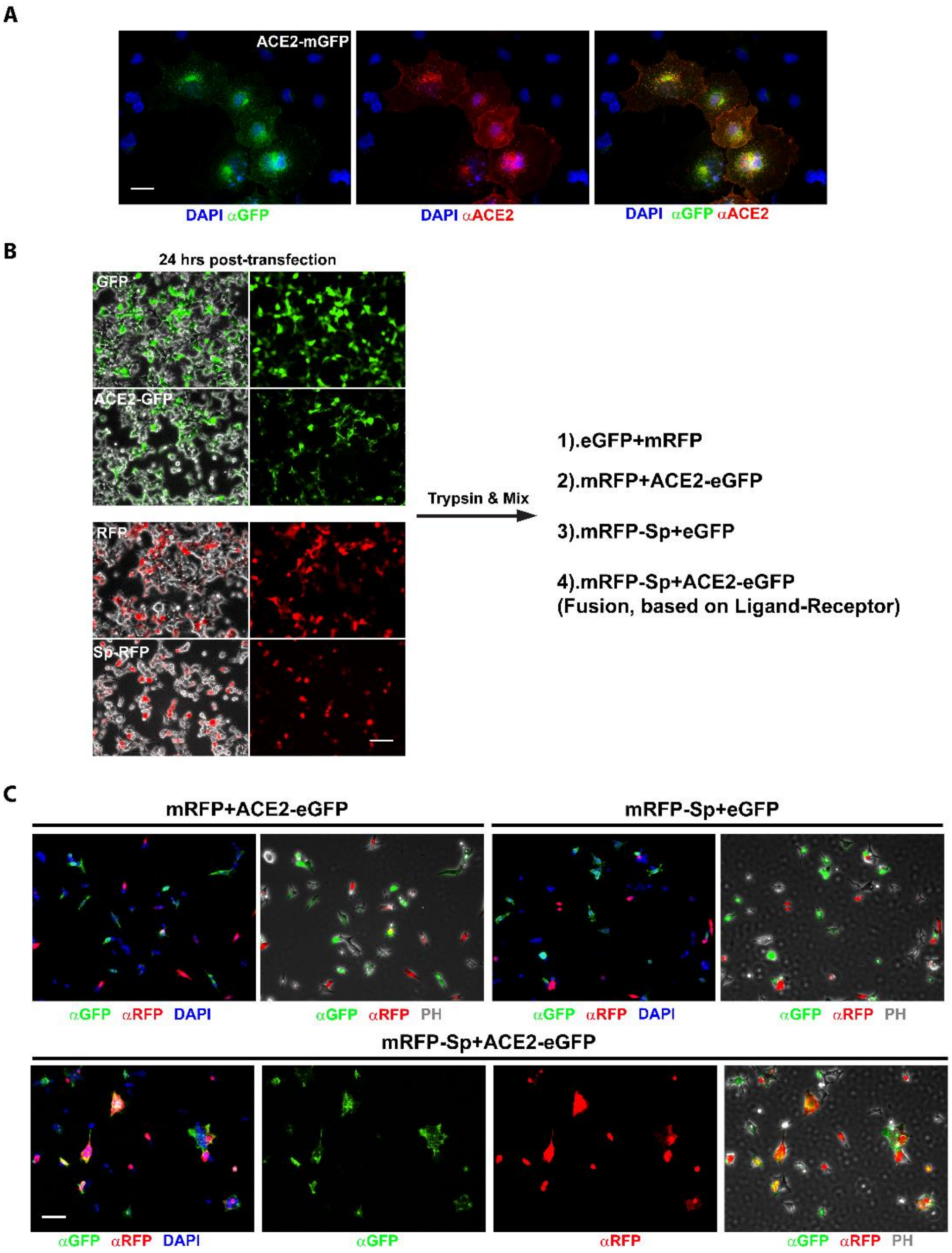

Supplementary Figure S3: Cell in fusion assay (A) ACE2-eGFP was transfected in COS7 cells and correctly confirmed expression pattern by ACE2 antibody. (B) 293T cells was separately transfected with eGFP, ACE2-eGFP, mRFP and mRFP-Sp. Overnight transfected cells were trypsinized and ACE2-eGFP was mixed with mRFP-Sp for cell in fusion assay. Other combinations were taken as control. (C) Cell in fusion was only observed in the combination of ACE2-eGFP and mRFP-Sp. No infusion phenome was observed in control combinations. Scale bar: 20μm.

Supplementary Tables:

Supplementary Table S1: The list of mutations that each variant possessed compared to the original spike protein sequence from the COVID-19 virus pathogen identified in Wuhan, China provided by the CDC (https://www.cdc.gov/coronavirus/2019-ncov/variants/variant-classifications.html).

Variant Name	Mutations identified
Alpha Variant	69del, 70del, 144del, E484K, S494P, N501Y, A570D, D614G, P681H, T716I, S982A, D1118H, K1191N
Beta Variant	D80A, D215G, 241del, 242del, 243del, K417N, E484K, N501Y, D614G, A701V
Gamma Variant	L18F, T20N, P26S, D138Y, R190S, K417T, E484K, N501Y, D614G, H655Y, T1027I
Delta Variant	T19R, G142D, 156del, 157del, R158G, L452R, T478K, D614G, P681R, D950N
Omicron Variant	A67V, 69-70del, T95I, 142-144del, Y145D, 211del, L212I, ins214EPE, G339D, S371L, S373P, S375F, K417N, N440K, G446S, S477N, T478K, E484A, Q493R, G496S, Q498R, N501Y, Y505H, T547K, D614G, H655Y, N679K, P681H, N764K, D796Y, N856K, Q954H, N969K, L981F
Note: Each of the numbers represent the residue location in reference to the spike protein sequence. A “del” refers to the deletion of the amino acid residue at the position. An “-ins” refers to the insertion of the amino acid sequence into that residue position (i.e., ins214EPE refers to the ‘E’, ‘P’, ‘E’ amino acids being inserted into residue 214). A mutation in the form of “amino acid 1 – residue – amino acid 2” refers to a substitution of the amino acid 1 with amino acid 2 at the residue position (i.e., L18F signifies L, or leucine, being substituted with F, or phenylalanine, at residue 18).	

Supplementary Table S2: The table represents the HADDOCK parameters of naturally occurring variants after refinement.

Variant Name	HADDOCK Score	Cluster Size	RMSD from the overall lowest-energy structure	Electrostatic Energy	Vander-Waals Energy	Desolvation Energy	Buried Surface area	Z-Score
**6M0J**	-121.5 ± 3.0	113	0.6 ± 0.3.	-314.3 ± 20.1	-64.6 ± 6.9	-17.8 ± 1.6	2047.9 ± 150.7	-1.7
**Original**	-129.5 ± 2.3	155	0.4 ± 0.3	-363.4 ± 28.0	-70.4 ± 6.3	-11.0 ± 1.2	2128.9 ± 47.5	-1.1
**Alpha Variant**	-111.4 ± 0.6	68	3.1 ± 0.3	-336.2 ± 69.4	-65.5 ± 9.9	-9.4 ± 2.2	2265.9 ± 106.1	-1.5
**Beta Variant**	-92.5 ± 9.6	14	18.2 ± 0.0	-311.1 ± 34.3	-59.4 ± 6.1	-6.2 ± 2.7	2041.4 ± 83.4	-1.0
**Gamma Variant**	-113.4 ± 6.9	6	0.5 ± 0.3	-294.4 ± 13.0	-75.8 ± 4.3	-21.7 ± 1.0	2389.0 ± 21.7	-1.9
**Delta Variant**	-95.4 ± 4.3	21	5.4 ± 0.6	-379.8 ± 39.3	-62.5 ± 7.5	-3.2 ± 5.1	2209.5 ± 174.2	-2.7
**Omicron Variant**	-112.8 ± 11.7	12	0.3 ± 0.2	-378.5 ± 61.8	-63.0 ± 7.4	-8.6 ± 1.6	2367.5 ± 111.8	-1.5

The HADDOCK score is protein-protein score, cluster size is the number of similar interaction conformations generated and clustered together by HADDOCK. RMSD (root mean square deviation) suggests how much deviation is the protein structure from the original confirmation. Van der waals energy is a result of comparatively weak electric forces. Electrostatic energy refers to electromagnetic force that occurs when a molecule is bearing static electric charge. Desolvation energy is required for the interacting proteins to substitute the water molecules on the binding surface. Z-score represents how many standard deviations the HADDOCK score of a given cluster is separated from the mean of all clusters.

Supplementary Table S3: The table represents the binding affinity and number of interactions between the trRosetta generated RBD's and the ACE receptor of 6M0J generated by the PRODIGY server.

Variant Name	ΔG (kcal/mol)	Kd (M) at 25.0 ℃	No. of Total ICs	IC's Charged-Charged	IC's Charged-Polar	IC's Charged-Apolar	IC's Polar-Polar	IC's Polar-Apolar	IC's Apolar-Apolar
**6M0J**	-13.9	6.4E-11	110	2	16	28	12	33	19
**Original**	-12.5	6.8E-10	112	2	17	26	16	31	20
**Alpha Variant**	-14.5	2.2E-11	115	3	12	37	10	30	23
**Beta Variant**	-13.1	2.5E-10	102	0	15	27	9	28	23
**Gamma Variant**	-13.9	6.4E-11	111	1	16	27	10	32	25
**Delta Variant**	-13.8	7.7E-11	113	1	20	26	10	32	24
**Omicron Variant**	-14.2	4.1E-11	106	8	11	23	7	30	27

Binding Affinity(ΔG) represents the firmness with which the two molecule binds with each other. Kd, dissociation energy; IC's, interfacial contacts

Supplementary Table S4: RMSD (root mean square deviation of atomic positions) score for trRosetta-generated mutated RBD models. The root-mean square deviation of atomic positions is the measure of the average distance (Å) between the atoms of superimposed proteins.

**Models**	**Root-Mean-Square Deviation (RMSD) Å**
Variant 1	0.682 for 1433 atoms
Variant 2	0.621 for 1451 atoms
Variant 3	0.634 for 1472 atoms
Variant 4	0.760 for 1454 atoms
Variant 5	0.690 for 1414 atoms
Variant 6	0.627 for 1471 atoms
Variant 7	0.777 for 1460 atoms
Variant 8	0.688 for 1410 atoms
Variant 9	0.632 for 1445 atoms
Variant 10	0.814 for 1453 atoms
Variant 11	0.733 for 1431 atoms

Supplementary Table S5: The table represents the HADDOCK parameters of all the manually mutated variants after refinement

Variants	HADDOCK Score	Cluster Size	RMSD (from the overall lowest-energy structure)	Vander- Waals Energy	Electrostatic Energy	Desolvation Energy	Buried Surface area	Z-score
**Variant 1**	-102.3 ± 7.8	12	1.0 ± 0.9	-81.0 ± 9.0	-180.3 ± 60.9	-20.7 ± 51.3	2050.8 ± 89.8	-1.3
**Variant 2**	-113.4 ± 8.0	139	1.2 ± 0.7	-75.4 ± 3.8	-303.2 ± 18.0	-15.7 ± 3.9	2101.4 ± 118.4	-1.7
**Variant 3**	-124.2 ± 3.1	43	0.4 ± 0.2	-70.4 ± 3.5	-309.1 ± 14.9	-17.4 ± 1.9	2057.9 ± 61.6	-2.8
**Variant 4**	-83.2 ± 6.7	10	5.5 ± 0.5	-69.4 ± 5.2	-194.3 ± 46.3	-4.4 ± 3.2	2132.5 ± 196.8	-1.4
**Variant 5**	-103.2 ± 9.3	17	17.0 ± 0.2	-67.6 ± 3.4	-290.5 ± 43.9	-4.5 ± 1.6	2168.1 ± 142.3	-1.3
**Variant 6**	-123.5 ± 1.2	192	1.4 ± 1.0	-74.3 ± 2.5	-296.5 ± 54.8	-16.5 ± 2.8	2168.7 ± 106.6	0.0
**Variant 7**	-111.8 ± 2.9	84	7.9 ± 0.1	-63.5 ± 8.7	-365.9 ± 23.1	-2.9 ± 2.2	2153.3 ± 72.4	-1.5
**Variant 8**	-127.1 ± 3.1	43	0.6 ± 0.4	-68.2 ± 5.1	-394.7 ± 31.4	-5.1 ± 4.5	2445.4 ± 15.4	-1.4
**Variant 9**	-50.9 ± 6.5	15	7.6 ± 0.2	-53.2 ± 13.1	-314.2 ± 43.4	-18.1 ± 4.4	2120.8 ± 139.7	-1.2
**Variant10**	-104.2 ± 5.0	10	0.4 ± 0.3	-66.1 ± 5.2	-377.8 ± 32.2	-3.7 ± 2.3	2390.3 ± 126.8	-1.5
**Variant11**	-100.2 ± 7.4	10	1.0 ± 0.9	-61.2 ± 6.8	-357.2 ± 16.7	3.7 ± 1.8	2335.6 ± 14.2	-2.5

Supplementary Table S6: The table represents the binding affinity and number of interactions between the trRosetta generated RBD's and the ACE receptor of 6M0J generated by the PRODIGY server.

Variant Name	ΔG (kcal/mol)	Kd (M) at 25.0 ℃	No. of Total ICs	IC's Charged-Charged	IC's Charged-Polar	IC's Charged-Apolar	IC's Polar-Polar	IC's Polar-Apolar	IC's Apolar-Apolar
**Variant 1**	-11.6	2.9E-09	67	3	5	21	3	18	17
**Variant 2**	-11.1	7.1E-09	70	4	7	20	5	17	17
**Variant 3**	-11.8	2.1E-09	69	3	8	20	5	21	12
**Variant 4**	-13.1	2.5E-10	115	2	17	34	11	26	25
**Variant 5**	-13.6	1.1E-10	101	8	11	24	6	26	26
**Variant 6**	-11.2	6.3E-09	67	3	8	20	5	18	13
**Variant 7**	-12.2	1.1E-09	103	2	17	22	14	30	18
**Variant 8**	-14.5	2.2E-11	101	8	12	22	5	30	24
**Variant 9**	-11.7	2.6E-09	75	7	6	23	7	19	13
**Variant 10**	-13.2	2.1E-10	120	4	17	35	11	25	28
**Variant 11**	-14.0	5.1E-11	109	11	13	30	5	23	27
